# Dioscin facilitates ROS-induced apoptosis via the p38-MAPK/HSP27-mediated pathways in lung squamous cell carcinoma

**DOI:** 10.7150/ijbs.45710

**Published:** 2020-09-02

**Authors:** Yinan Yao, Luyun Cui, Jiani Ye, Guangdie Yang, Guohua Lu, Xiaomei Fang, Zhu Zeng, Jianying Zhou

**Affiliations:** Department of Respiratory Medicine, The First Affiliated Hospital, College of Medicine, Zhejiang University, Hangzhou, China.

**Keywords:** lung SCC, dioscin, cell apoptosis, ROS, p38-MAPK

## Abstract

Lung squamous cell carcinoma (SCC) is one of the deadliest cancers both in China and worldwide. To date, the efficacy of lung SCC treatments is limited. Recent studies have elucidated the powerful anti-tumour role of dioscin in different human cancers. Here, our study aims to investigate the effect of dioscin on lung SCC and its underlying mechanism. First, we found that dioscin not only inhibited cell proliferation and cell migration and induced cell apoptosis in lung SCC cells but also suppressed tumour growth in tumour-bearing mice. Furthermore, we noted that the accumulation of intracellular reactive oxygen species (ROS) was triggered by dioscin in lung SCC cells, leading to the phosphorylation of HSP27 through p38-MAPK and consequent cell apoptosis. The activation of p38-MAPK/HSP27 induced by the p38-MAPK activator Anisomycin enhanced the apoptosis of lung SCC cells, while the ROS inhibitor N-acetyl-L-cysteine (NAC) and the p38-MAPK inhibitor SB203580 both attenuated dioscin-mediated cell apoptosis. Moreover, NAC suppressed the activation of p38-MAPK/HSP27 that induced by dioscin. In conclusion, these results confirm that dioscin facilitates ROS-induced apoptosis via the p38-MAPK/HSP27-mediated pathway in lung SCC.

## Introduction

According to global cancer statistics, lung cancer ranks first in terms of incidence and cancer-related mortality worldwide 1, 2. Lung squamous cell carcinoma (SCC) is tobacco-related and accounts for approximately about 20-30% of lung cancers 3, 4. The rapid development of targeted therapy promotes precision cancer therapy 5. However, in lung SCC, with the lack of effective targetable mutations, combination chemotherapy is still the standard therapy and has limited benefit 3. Hence, it is essential to seek potential targets and novel therapeutic options for the treatment of lung SCC.

Recently, dioscin, a steroidal saponin isolated from the roots of Dioscorea plants, has shown promising medicinal value in regulating inflammation, immunity, lipidemic and tumour development 6. Interestingly, previous studies also confirmed the protective effect of dioscin against doxorubicin-induced cardiotoxicity 7. Notably, abundant evidence suggests dioscin can effectively inhibit different cancers, including lung cancer, pancreatic cancer, gastrointestinal cancer and breast cancer 6, 8-11. Dioscin exerts its anticancer effect by inducing cell cycle arrest and regulating TIGAR-mediated autophagy in liver cancers 9, 10. Moreover, dioscin has been confirmed to induce DNA damage and cell apoptosis and to inhibit cell proliferation and cell invasion in human lung cancer cells 11, 12. Although the application of dioscin has been assessed in several tumours, the effect of dioscin on lung SCC and the underlying mechanism are still unknown.

Reactive oxygen species (ROS), a sub-product of oxidative energy metabolism, is vital in regulating cell functions, such as proliferation, differentiation, migration and death 13. Recent studies confirmed that the accumulation of intracellular ROS caused by external stimuli, including dioscin, can trigger cell apoptosis through relevant signalling pathways 13-15. In human oesophageal cancer cells, dioscin inhibits the expression of peroxiredoxins to induce ROS-mediated cell apoptosis 15. Moreover, dioscin upregulates ROS levels to accelerate DNA damage, mitochondrial dysfunction and subsequent cell apoptosis in other cancers 16, 17. Hence, we consider that the anti-tumour effect of dioscin on cell apoptosis is associated with ROS.

Intracellular ROS is also regarded as a significant modulator of the p38-MAPK signalling pathway in cell apoptosis in various cancers 14, 18-20. For example, in lung cancer cells, the accumulation of intracellular ROS caused by paraquat activates p38-MAPK and thus modulates the mitochondrial apoptotic pathway 18. Dioscin is reported to suppress the growth of human laryngeal cancer cells by activating p38-MAPK-mediated apoptosis 21. Thus, we reasonably believe that dioscin-induced cell apoptosis may be related to p38-MAPK. Heat shock protein 27 (HSP27) is a downstream target of p38-MAPK and participates in proteasome-mediated protein degradation, cytoskeleton remodelling and apoptosis 22, 23. Although some previous studies have indicated that HSP27 phosphorylation contributes to p38-MAPK-induced apoptosis, it may play a cytoprotective role under certain conditions 24, 25. We still need further exploration of the relationship between dioscin-mediated cell apoptosis and the p38-MAPK/HSP27 signalling pathway.

We hypothesized that dioscin could induce cell apoptosis through the ROS-mediated p38-MAPK/HSP27 signalling pathway in lung SCC cells. To confirm our hypothesis, we evaluated the effect of dioscin on lung SCC both *in vivo* and *in vitro* and then elucidated the underlying signalling pathways.

## Materials and Methods

### Cell lines and Reagents

Human lung SCC cell line SK‐MES‐1 and human bronchial epithelial cell line HBE were bought from the Committee on Type Culture Collection of the Chinese Academy of Sciences (Shanghai, China). And NCI‐H520 was a gift from Dr Ying (Department of Respiratory Diseases, Sir Run Run Shaw Hospital, Zhejiang University, Hangzhou, China). All cells were cultured in RPMI-1640 (Hyclone, Logan, UT, USA) with 10% fetal bovine serum (FBS, Gibco BRL Co., Ltd., Houston, TX, USA), 100 U/ml penicillin and 100 U/ml streptomycin (Solarbio, Beijing, China), at 37℃ in 5% CO_2_. Dioscin with the purity of over 98% was purchased from Shanghai Tauto Biochemical Technology Co., Ltd (Shanghai, China). Dioscin was dissolved in dimethyl sulfoxide (DMSO) (Sangon Biotech, Shanghai, China) at a concentration of 20 mM and stored at -20 °C. The final concentration of DMSO in all treatments was less than 0.1%. The p38 inhibitor SB203580 and the p38 activator Anisomycin were purchased from Selleck Chemicals (Houston, TX, USA), while reactive oxygen species inhibitor antioxidant N-Acetyl-L-cysteine (NAC) was purchased from Sigma‐Aldrich (St. Louis, MO, USA).

### Cell viability assay

Lung SCC cells and HBE cells were seeded in 96-well plates. Then cells were treated with different concentrations of dioscin for 24 h and 48 h. After that, cell counting kit-8 (Dojindo Laboratories, Tokyo, Japan) were added into cells with 1-2 h incubation and the OD value was read by SpectraMax i3x Muliti-Mode Microplate Reader (Molecular Devices, San Francisco, CA, USA) at 450 nm.

### Colony formation assay

NCI-H520 and SK-MES-1 cells were seeded in 6-well plates at a density of 500 cells/well. After overnight adhesion, cells were treated with different concentrations of dioscin (0, 1.25, 2.5, 5 μM) for 7-10 days. Then, cells were stained with 0.1% crystal violet and imaged.

### Wound healing assay

When NCI-H520 and SK-MES-1 cells reached 80% confluency in six-well plates, the cells were wounded with a sterile 10 μl pipette tip on the cell monolayers and washed with serum-free medium to remove detached cells. Next, the cells were treated with dioscin (0, 1.25 and 2.5 μM) for 24 h and 48 h. Finally, the images of wound gap were taken using microscope (Olympus, Tokyo, Japan).

### Transwell assay

SK-MES-1 and NCI-H520 cells were starved overnight and then cultured in the upper chamber (24-well transwell chambers, 8 μm pore size, Corning, NY, USA) with serum-free medium, while high-serum medium (10% FBS) containing dioscin was added to the lower chamber. After 48 h of incubation at 37 °C, cells were fixed with methanol and stained with 0.1% crystal violet. Finally, images were taken using microscope (Olympus, Tokyo, Japan).

### Cell apoptosis assay

Lung SCC cells and HBE cells were harvested and a FITC Annexin V Apoptosis Detection Kit (BD Pharmingen, NJ, USA) was used to detect cell apoptosis according to the manufacturer's protocol. Briefly, cells were washed and resuspended in binding buffer. Then, cells were stained with 5 μl of FITC Annexin V and 5 μl of propidium iodide (PI) followed by incubation for 15 mins at room temperature in the dark. Finally, cell apoptosis was analyzed by BD FACSVerse (BD Biosciences, San Jose, CA, USA).

### Analysis of mitochondrial ΔΨm

The mitochondrial ΔΨm was detected by JC-1 Mitochondrial Membrane Potential Assay Kit (Yeasen Biotech, Shanghai, China) according to the manufacturer's instructions. After different treatments, JC-1 working solution was added to the cells and incubated at 37 °C for 20 mins. Next, cells were washed twice with JC-1 staining buffer and analysed by BD FACSVerse (BD Biosciences, San Jose, CA, USA). And when ΔΨm is reduced, red fluorescence decreases and green fluorescence increases. Thus, ΔΨm variations can be reflected by the ratio of green/red fluorescence intensity.

### Analysis of intracellular ROS

The intracellular ROS of cells were detected by Reactive Oxygen Species Assay Kit (Beyotime, Shanghai, China) with 2',7'‑dichlorodihydrofluorescein diacetate (DCFH‑DA) according to the method published previously with fine-turning^14^. Briefly, cells were harvested, washed with PBS and incubated with DCFH‑DA (0.5 μM) in the dark for 3 mins at 37 °C. After washing with PBS, the samples were analysed for the fluorescence of DCF by BD FACSVerse (BD Biosciences, San Jose, CA, USA).

### Western blotting analysis

Cells and tissues were lysed in RIPA lysis buffer (Beyotime, Shanghai, China). Then, the protein was separated by SDS-PAGE, transferred to PVDF membranes (Millipore, Bedford, MA, USA) and probed with primary antibodies overnight at 4 ℃. After incubated with the corresponding secondary antibodies for 1 h, the densities of bands were detected by ECL Chemiluminescence Kit HRP (FDbio, Hangzhou, China). Antibodies against Bcl2, Bax, cleaved caspase-3, cleaved PARP, p38, p-p38 (Thr180/Tyr182), E-cadherin, N-cadherin and Vimentin were purchased from Cell Signalling Technology (Danvers, MA, USA). Antibodies against HSP27 and p-HSP27 (Ser15) were purchased from Diag Biotechnology (Hangzhou, China). The antibody against GAPDH was obtained from Beyotime (Shanghai, China).

### Xenograft model experiment

Female BALB/c-nude mice (3-4 weeks) were purchased from Shanghai Experimental Animal Center (Chinese Academy of Sciences, Shanghai, China). 8×10^5^ suspended NCI-H520 cells (in 100 μl PBS) were injected into the right back of each mouse. When tumour volumes reached approximately 50-100 mm^3^, the mice were randomized into 2 groups (6 mice per group). Each group of mice was given with 0.5% Sodium carboxymethylcellulose (Sangon Biotech, Shanghai, China) or dioscin (80 mg/kg/day) by gavage for 12 days. Tumours were measured every 3 days using a vernier calliper, and the tumour volume was calculated by the following formula: V = (Length *Width^2^)/2. At the endpoint, mice were sacrificed and the tumours were collected for further analysis. The experiments were performed according to the Regulations for the Administration of Affairs Concerning Experimental Animals and were approved by the Experimental Animal Ethics Committee of Zhejiang University.

### TUNEL assay

Apoptosis in tissues was detected by One Step TUNEL Apoptosis Assay Kit (Beyotime, Shanghai, China), according to the instruction provided with the kit. Briefly, the tissue sections were dewaxed, rehydrated, and permeabilized with 20 μg/ml proteinase K solution for 20 mins at 37 °C. After washed with PBS, the tissue sections were incubated with TUNEL reaction mixture in the dark for 1 h at 37 °C. The nuclei were stained with 4′,6-diamidino-2-phenylindole (DAPI). After washed with PBS, the tissue sections were mounted with antifade mounting medium (Beyotime, Shanghai, China) and images were taken using microscope (Olympus, Tokyo, Japan).

### Statistical analysis

All data were presented as the mean ± SD of three independent experiments. The statistical significance was assessed by Student's t-test for two groups or one-way ANOVA for multiple groups using Prism 6.04 software (GraphPad Software Inc., San Diego, CA, USA). *p<0.05, **p<0.01, ***p<0.001 are determined as significance.

## Results

### Dioscin inhibited cell proliferation in lung SCC cells

**Figure 1:**
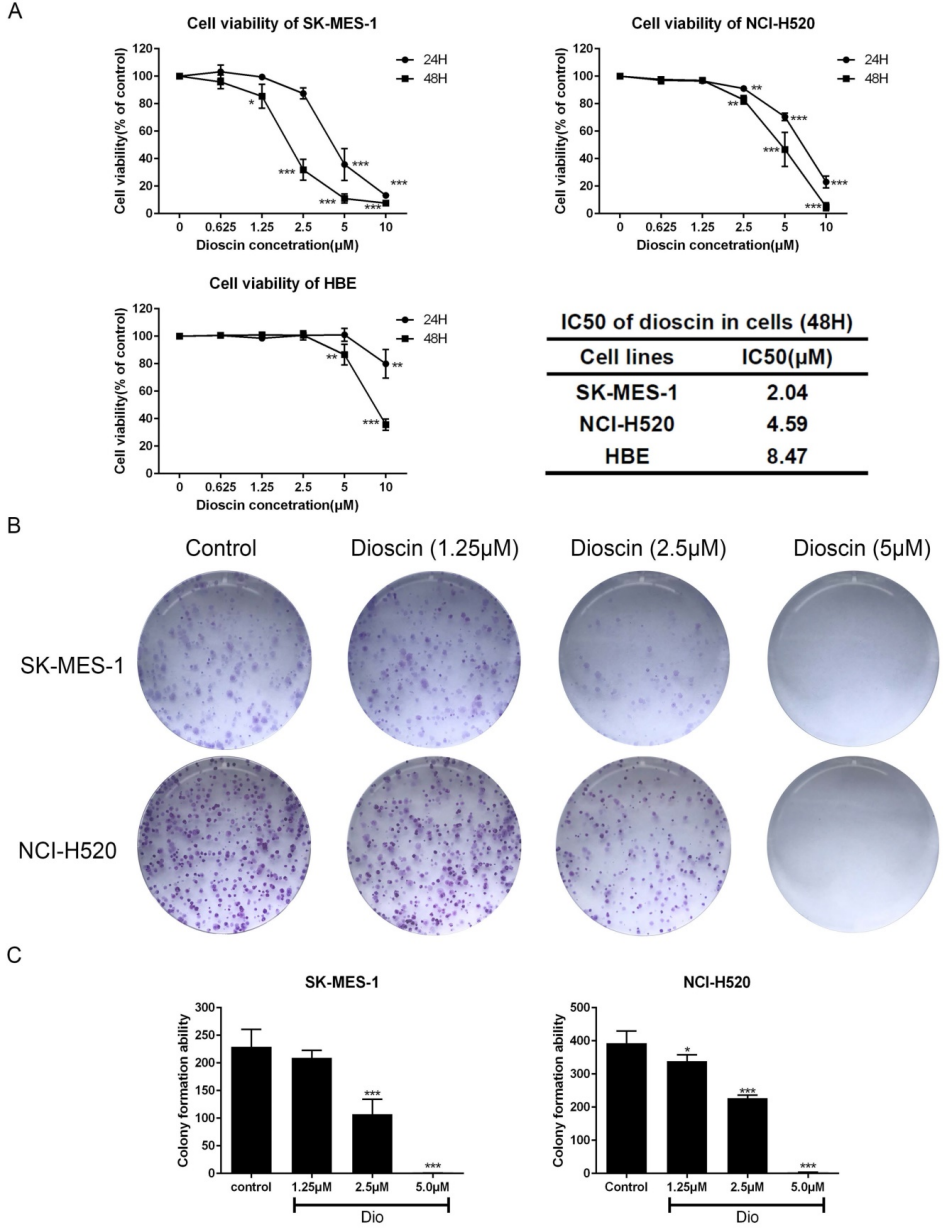
Dioscin inhibited cell proliferation in lung SCC cells. (A) SK-MES-1, NCI-H520 and HBE cells were treated with Dio (0, 1.25, 2.5, 5, 10 µM) for 24 h and 48 h. Cell viability was determined by CCK-8 assay, which was performed in triplicate with three independent experiments. The IC50 of dioscin at 48 h in each cell line was calculated. (B, C) Dioscin suppressed SK-MES-1 and NCI-H520 cells colony formation. Cells were treated with Dio (0, 1.25, 2.5, 5 µM). After 7-10 days, the cells were stained with 0.1% crystal violet. The data are representative of three independent experiments and are presented as the mean ± SD. Significant differences compared with the control are indicated by *p<0.05, **p<0.01, and***p<0.001.

Lung SCC cells and HBE cells were treated with dioscin (0, 1.25, 2.5, 5, 10 μM) for 24 and 48 h. As shown in Fig. 1A, the viability of lung SCC cells was significantly inhibited by dioscin in a dose-dependent manner, with IC50 values of 4.59 μM (NCI-H520) and 2.05 μM (SK-MES-1) at 48 h. The IC50 value of HBE cells after 48 h of treatment was 8.47 μM, indicating that the effect of dioscin was more potent in lung SCC cells than in HBE cells (Fig. 1A). A colony formation assay was performed to determine the effect of dioscin on lung SCC cell proliferation. We found that dioscin substantially decreased the colony numbers in both NCI-H520 and SK-MES-1 cells (Fig. 1B and 1C).

### Dioscin suppressed cell migration in lung SCC cells

**Figure 2:**
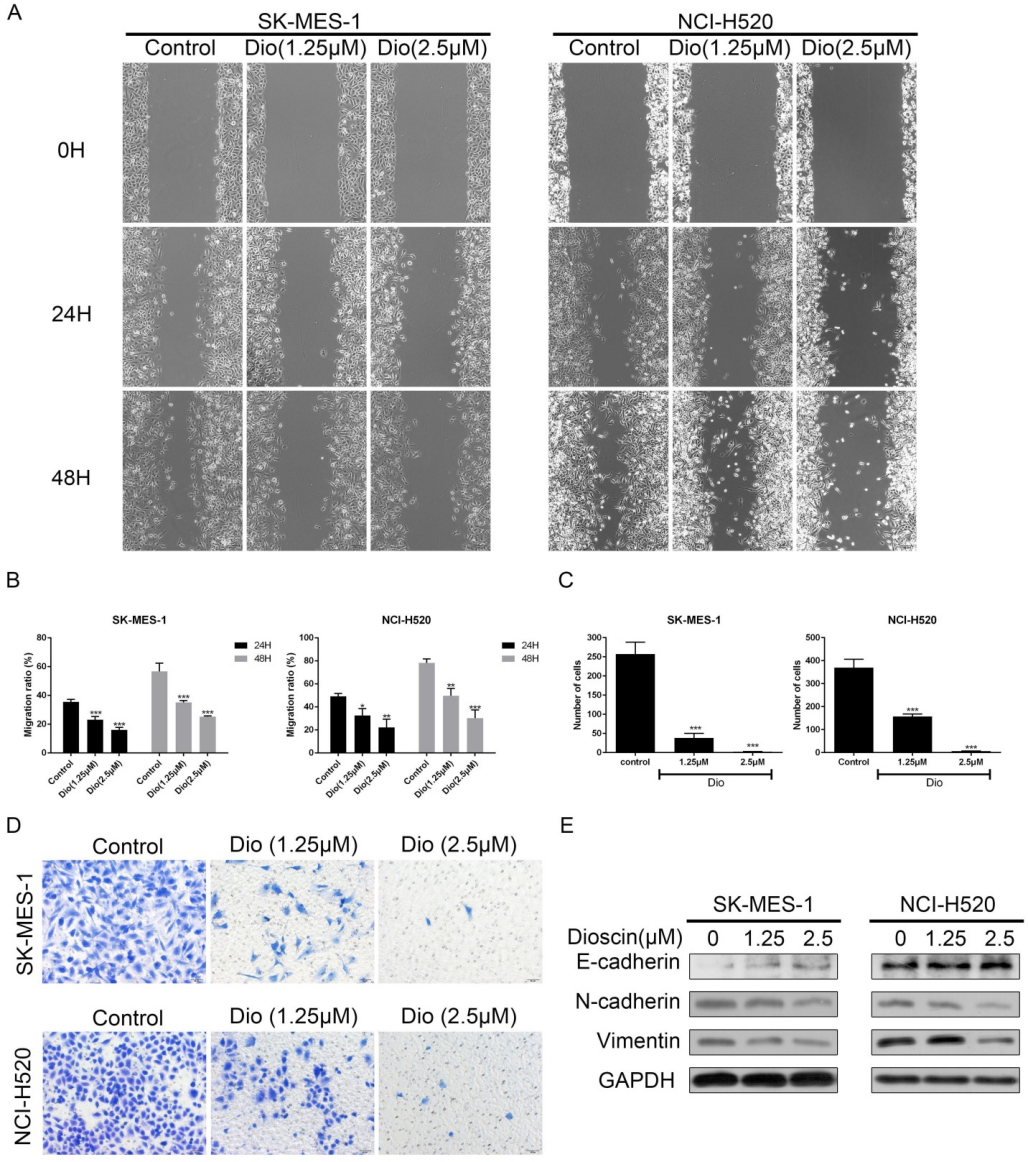
Dioscin suppressed the cell migration of lung SCC cells. SK-MES-1 and NCI-H520 cells were treated with Dio (0, 1.25, 2.5 µM) for 48 h. The effect of dioscin on cell migration was assessed by wound healing assay (scale bar=100 µm) (A, B) and Transwell assay (scale bar=50 µm) (C, D). The data are representatives of three independent experiments and presented as the mean ± SD. Significant differences compared with the control are indicated by *p<0.05, **p<0.01, and***p<0.001.(E) The expression levels of E-cadherin, N-cadherin and Vimentin were analysed by western blot.

Wound healing and Transwell assays were used to determine the impact of dioscin on cell migration. As shown in Fig. 2A and 2B, dioscin treatment (1.25 and 2.5 μM) resulted in slower rates of wound healing. In addition, dioscin markedly reduced the numbers of NCI-H520 and SK-MES-1 cells moving to the lower chamber (Fig. 2C and 2D). Epithelial-to-mesenchymal transition (EMT) is a program which enhances the migration of tumour cells 26. So, we also detected the expression changes of EMT-related proteins. And we found that dioscin mildly down-regulated the expression levels of N-cadherin and Vimentin, and up-regulated the expression levels E-cadherin (Fig. 2E). Therefore, dioscin could suppress the migration of lung SCC cells.

### Dioscin induced cell apoptosis in lung SCC cells

**Figure 3:**
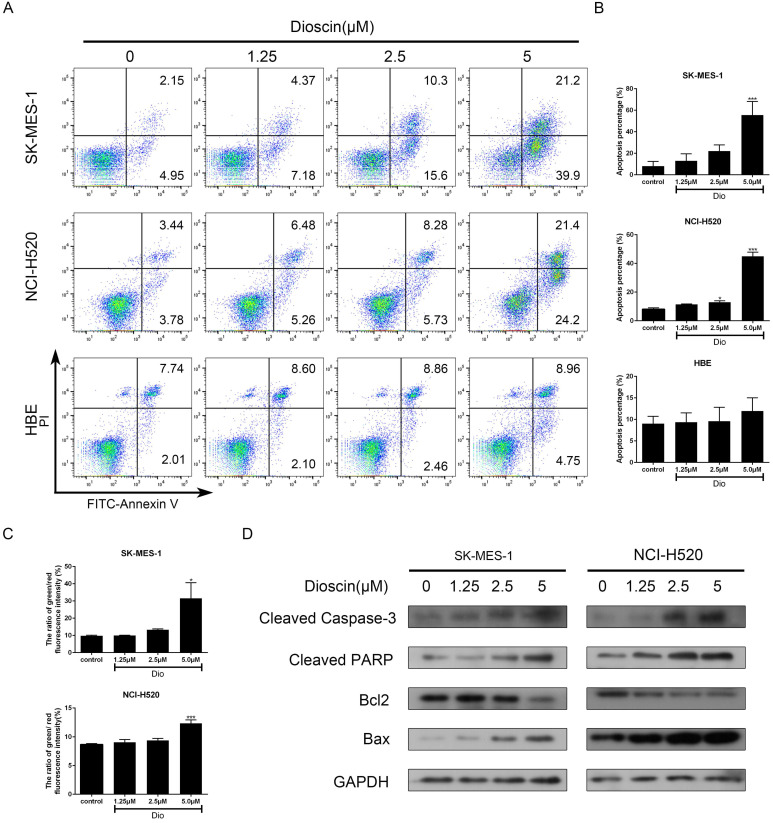
Dioscin induced cell apoptosis in lung SCC cells. NCI‐H520, SK‐MES‐1 and HBE cells were treated with dioscin (0, 1.25, 2.5, 5 µM) for 48 h. (A, B) Cell apoptosis was measured by flow cytometry. (C) The changes in ΔΨm were monitored by JC-1 staining and the ratio of green/red fluorescence intensity was used to calculate mitochondrial depolarization. Data are the mean ± SD of triplicate samples. Significant differences compared with the control are indicated by *p<0.05, **p<0.01, and***p<0.001. (D) The expression levels of cleaved caspase-3, cleaved PARP, Bax and Bcl-2 were analysed by western blot.

NCI-H520, SK-MES-1 and HBE cells were treated with dioscin for 48 h and cell apoptosis was detected by PI/FITC- Annexin V double staining. Our data revealed that dioscin had a significant dose-dependent effect on apoptotic inducement in lung SCC cells, while the effect in HBE cells was not obvious (Fig. 3A and 3B). A reduction in ΔΨm often marked the early stage of mitochondrial apoptosis. Compared with the control group, dioscin treatment increased the ratio of green/red fluorescence intensity in both NCI-H520 and SK-MES-1 cells (Fig. 3C). Then, we detected the expression of B cell lymphoma 2 (Bcl2) and its family members to explore how dioscin influences cell apoptosis. We found that dioscin downregulated the expression of Bcl2 and upregulated the expression of Bax (Fig. 3D). Moreover, caspase‐3 and its downstream target PARP, which indicate the point at which the extrinsic and intrinsic apoptotic pathways merge, were obviously activated with dioscin treatment (Fig. 3D).

### The p38-MAPK/HSP27 signalling pathway was involved in apoptosis induced by dioscin

**Figure 4:**
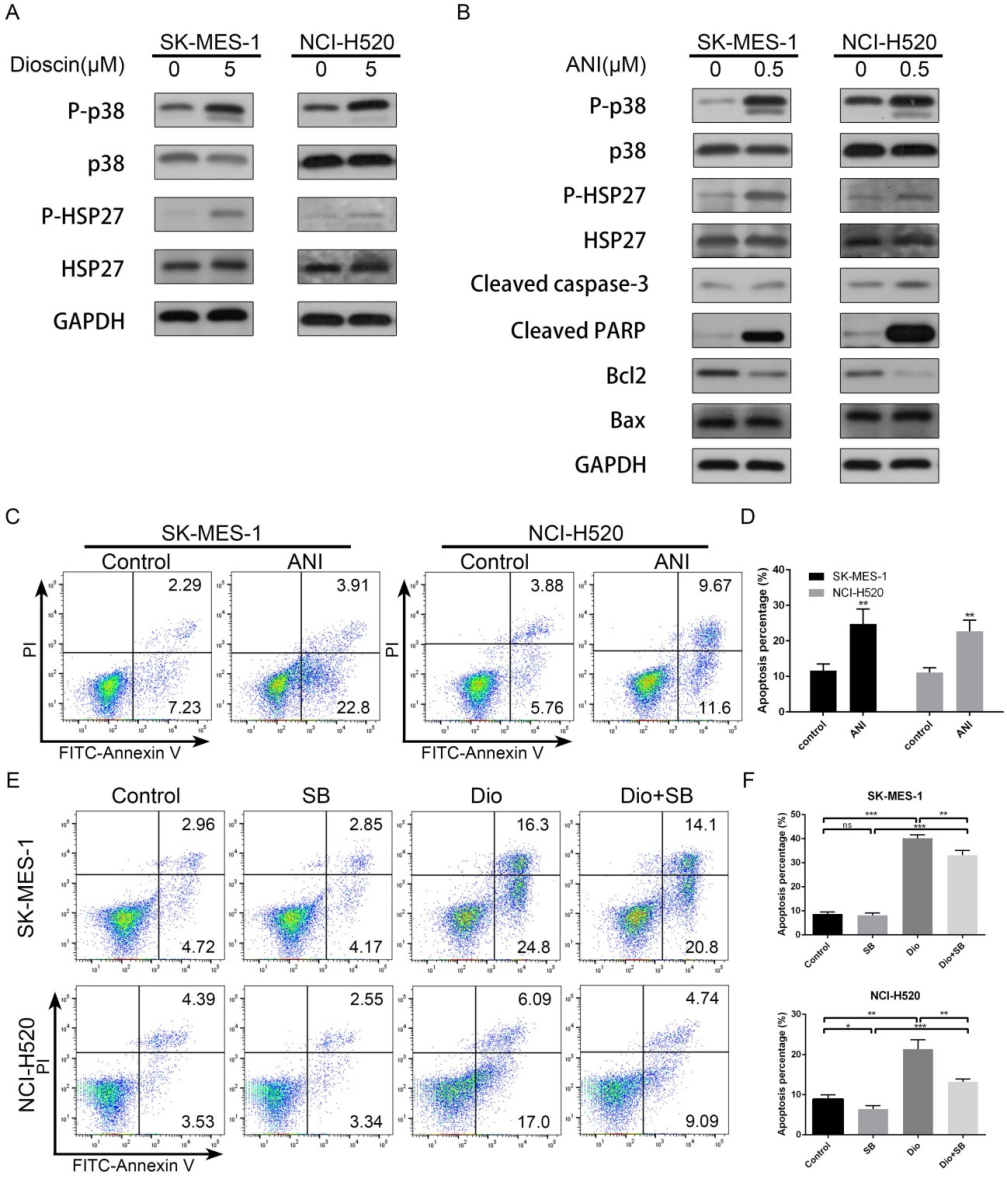
The p38-MAPK/HSP27 signalling pathway was involved in dioscin-induced apoptosis. (A) NCI‐H520 and SK‐MES‐1 cells were treated with dioscin (5 µM) for 48 h. The levels of p-p38, p38, p-HSP27 and HSP27 were analysed by western blotting. (B, C) NCI‐H520 and SK‐MES‐1 cells were treated with Anisomycin (0.5 µM) for 24 h. Then, cell apoptosis was measured by flow cytometry, and the expression levels of p-p38, p38, p-HSP27, HSP27, Bax, Bcl2, cleaved caspase-3 and cleaved PARP were analysed by western blotting. (D) NCI‐H520 and SK‐MES‐1 cells were pretreated with SB203580 (5 µM) for 2 h before exposure to dioscin for 48 h. Then, cell apoptosis was measured by flow cytometry. Data are presented as the mean ± SD of triplicate samples. *P < 0.05, **P < 0.01, and ***P < 0.001 vs control.

We further evaluated the effect of dioscin on the p38-MAPK/HSP27 pathway in lung SCC cells. As shown in Fig. 4A, we found that dioscin clearly increased the levels of p-p38 and p-HSP27, while the levels of total p38 and HSP27 were not obviously changes in NCI-H520 and SK-MES-1 cells. In addition, to ascertain whether the p38-MAPK/HSP27 signalling pathway was involved in dioscin-induced apoptosis, we pre-treated the cells with p38-MAPK activator Anisomycin and p38-MAPK inhibitor SB203580. Anisomycin pre-treatment activated p38 and HSP27 and induced apoptosis (Fig. 4B and 4C), while SB203580 partly reversed dioscin-induced apoptosis (Fig. 4D). In conclusion, the p38-MAPK/HSP27 signalling pathway promoted dioscin-mediated apoptosis.

### Intracellular ROS accumulation was a pivotal event in dioscin-induced apoptosis

**Figure 5:**
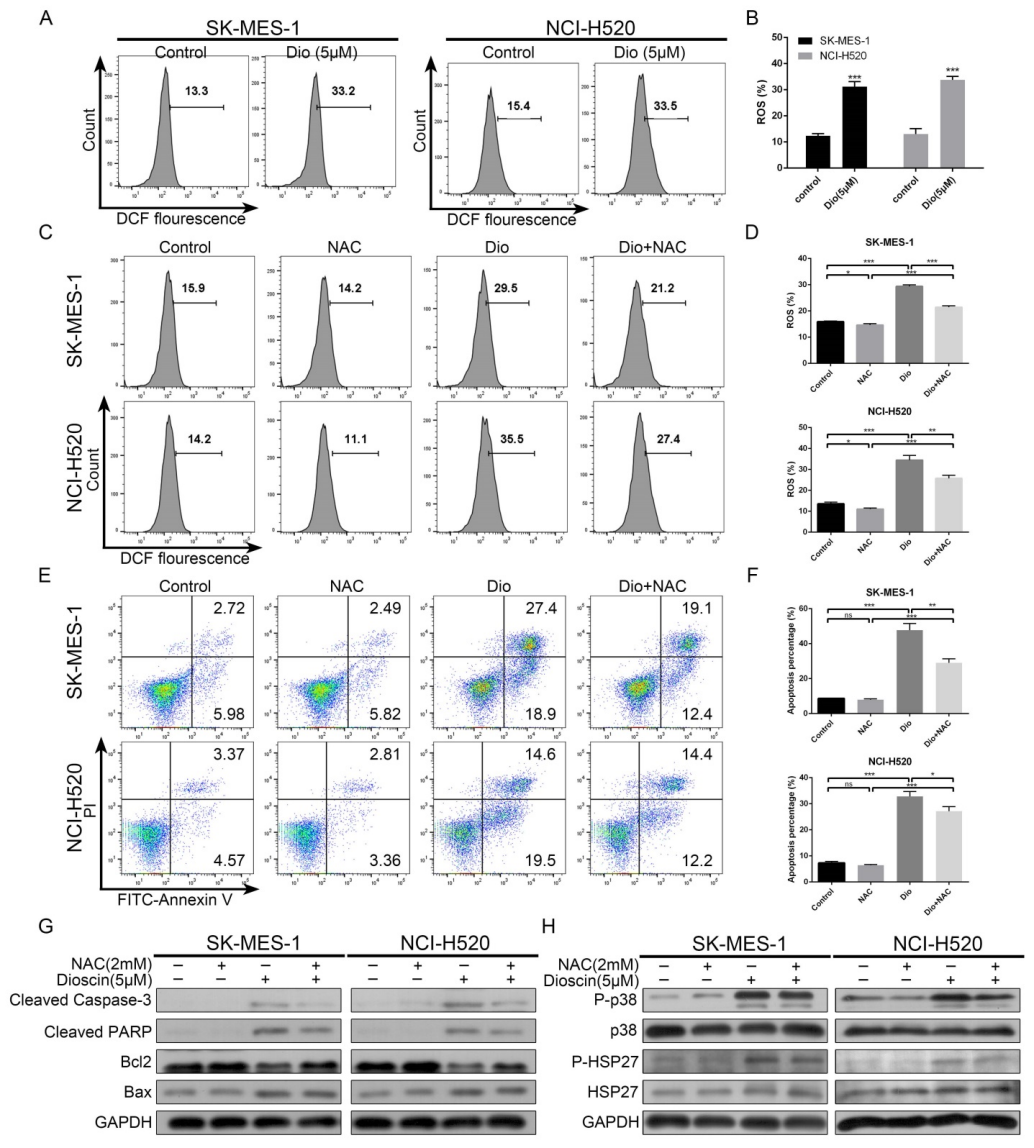
Intracellular ROS accumulation was a pivotal event in dioscin-induced apoptosis. (A, B) NCI‐H520 and SK‐MES‐1 cells were treated with dioscin (5 µM) for 48 h. The intracellular ROS levels were measured by flow cytometry. Data are presented as the mean ± SD of triplicate samples. *p < 0.05, **p < 0.01, and ***p < 0.001 vs control. (C-H) NCI‐H520 and SK‐MES‐1 cells were pretreated with NAC (2 mM) for 24 h before exposure to dioscin for 48 h. Then, cell apoptosis and intracellular ROS levels were measured by flow cytometry. Data are presented as the mean ± SD of triplicate samples. *p < 0.05, **p < 0.01, and ***p < 0.001 vs control. The expression levels of cleaved caspase 3, cleaved PARP, Bax, Bcl2, p-p38, p38, p-HSP27 and HSP27 were analysed by western blotting.

To demonstrate whether ROS was involved in dioscin-induced lung SCC cell apoptosis, we analysed the intracellular ROS generation in lung SCC cells after dioscin treatment. As shown in Fig. 5A and 5B, dioscin elevated intracellular ROS levels in NCI-H520 and SK-MES-1 cells. Then, NAC, an ROS inhibitor antioxidant, was used to pre-treat lung SCC cells. We found NAC markedly attenuated dioscin-induced ROS accumulation and cell apoptosis in lung SCC cells (Fig. 5C-G). Moreover, NAC combined with dioscin decreased the levels of p-p38 and p-HSP27 compared to those with dioscin treatment alone (Fig. 5H). Therefore, we considered that dioscin may trigger the ROS-mediated p38-MAPK/HSP27 signalling pathway, leading to cell apoptosis.

### Dioscin exerted an anti-tumour effect in NCI-H520 xenograft models

**Figure 6:**
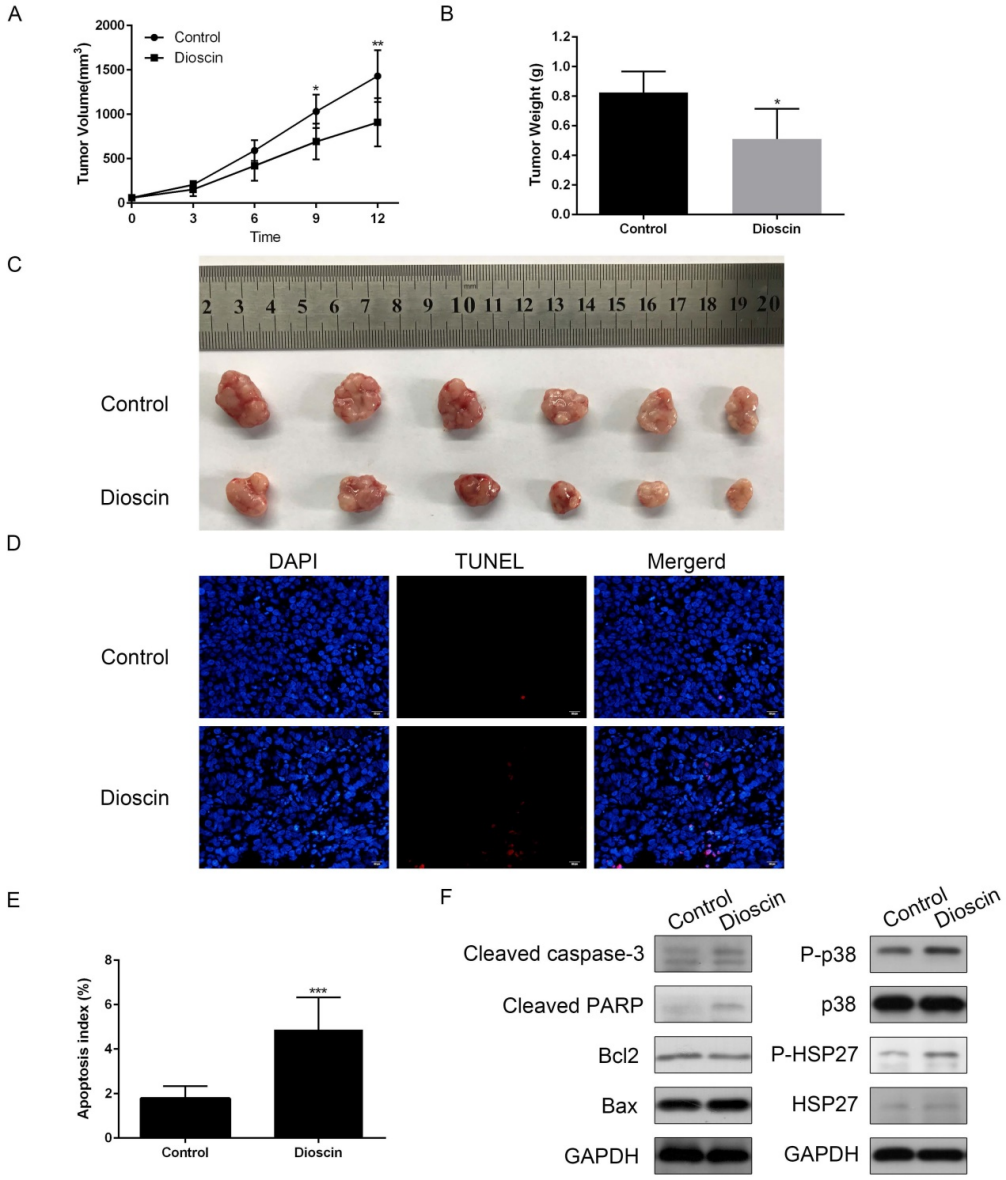
*In vivo* anti-tumour effects of dioscin in xenograft models. NCI-H520 cells were subcutaneously injected into the backs of BALB/c nude mice. When the tumour reached a volume of 50-100 mm^3^, mice were divided into two groups and orally treated with vehicle or dioscin (80 mg/kg/d) for 12 days (n=6). (A) The tumour volumes of the mice were determined every three days after the onset of treatment. (B, C) On day 12, the tumours were carefully dissected from the mice and the weights of tumours was measured. (D, E) Apoptotic cells in tumour samples were detected by TUNEL assay (scale bar=20 µm). Data are presented as the mean ± SD. Significant differences compared with the control are indicated by *p<0.05, **p<0.01, and***p<0.001. (F) The harvested tumours were subsequently lysed and western blot analysis was performed for cleaved caspase-3, cleaved PARP, Bax, Bcl2, p-p38, p38, p-HSP27, HSP27 and GAPDH expression.

In the present study, we constructed NCI-H520 xenograft models to confirm the anti-tumour effect of dioscin *in vivo*. As shown in Fig. 6A, there was a significant reduction in tumour volume in dioscin-treated mice compared with vehicle-treated control mice at day 12. At the endpoint of the experiment, the mice were sacrificed. Tumours were harvested, and the tumour weights were measured. The weight of NCI-H520 tumours in dioscin-treated mice was lower than that in control mice (Fig. 6B and 6C). Then, we detected apoptosis in the xenograft tumours. The TUNEL assay showed that the number of apoptotic cells was higher in the dioscin treatment group than in the control group (Fig. 6D and 6E). Moreover, the expression levels of cleaved caspase-3 and cleaved PARP in the dioscin treatment group were also increased (Fig. 6F). To evaluate whether the inhibition of lung SCC tumour xenograft growth by dioscin was associated with the p38-MAPK/HSP27 signalling pathway, we determined the levels of p38, p-p38, HSP27, p-HSP27 in tumour samples by western blotting. Here, we found that dioscin treatment activated the p38-MAPK/HSP27 signalling pathway in tumour tissue and these results were similar to the results *in vitro* (Fig. 6F).

## Discussion

Though multimodality therapies have been developed, lung SCC is still a highly aggressive disease with limited benefit from current treatments. Therefore, the identification of novel adjuvant treatments for lung SCC is still urgent. Recent studies have shown the powerful anti-tumour effect of dioscin in different cancer 6. In lung cancer, it has been proven that dioscin inhibits TGFβ1-induced cell migration and invasion 27. A previous study also found that dioscin suppresses lung cancer growth by inducing DNA damage and activating mitochondrial apoptosis 11. Moreover, targeted liposomes loaded with both daunorubicin and dioscin inhibit metastasis of lung cancer 28. More importantly, dioscin overcomes tyrosine kinase inhibitor resistance by down-regulating phosphatase SH2 domain-containing phosphatase-2 (SHP2) expression in EGFR-mutated lung adenocarcinoma cells 29. However, few studies have focused on the antineoplastic activity of dioscin in lung SCC. The present study indicated that dioscin inhibited lung SCC growth both *in vitro* and *in vivo*. The anti-tumour effects of dioscin were mainly associated with signalling pathways involving the accumulation of ROS and the activation of p38-MAPK and its downstream target HSP27.

Uncontrolled cell growth and migration are two of the biological behaviours of malignant cells that cause poor prognosis in patients with lung cancer 12, 27. Our results showed that dioscin obviously inhibited cell proliferation and cell migration in lung SCC cells, which was similar to the results of previous studies 6, 8, 12. NCI-H520 xenograft model experiments also showed the significant anti-tumour activity of dioscin. The tumour volume and tumour weight of mice receiving dioscin treatment were apparently decreased compared to those of control mice. In accordance with previous studies, we found that dioscin induced cell apoptosis in lung SCC cells, regardless of whether the apoptosis was in the early stage or late stage, with a marked upregulation expression of cleavage of caspase-3 and PARP 6, 8, 17. Moreover, the results of xenograft models verified the pro-apoptotic effect of dioscin *in vivo*. Importantly, our study further indicated that dioscin-induced cell apoptosis was related to ROS.

It has been reported that the regulation of intracellular redox status by ROS is related to apoptosis by influencing intracellular signalling pathways 30, 31. Fluctuations in intracellular ROS levels induced by dioscin cause damage to cellular structures, which accelerating apoptosis 15. ROS generation is explicated to induce apoptosis in human lung SCC cells 31. In the present study, we noted that the accumulation of intracellular ROS was triggered by dioscin in lung SCC cells. In addition, the ROS scavenger NAC exerted strong effects not only by decreasing dioscin-induced intracellular ROS but also by attenuating dioscin-induced apoptosis. Hence, we elucidated dioscin-induced apoptosis in lung SCC cells by upregulating intracellular ROS levels.

ROS has been demonstrated to regulate p38-MAPK, which extensively participates in the regulation of cell apoptosis, proliferation and differentiation 14, 32. In colon cancer cells, dioscin activates the p38-MAPK signalling pathway to induce ROS-mediated apoptosis 17. However, some controversial evidence has proven that inactivation of p38-MAPK inhibits tumorigenesis 33, 34. HSP27 is one of the downstream targets of p38-MAPK and high levels of HSP27 enhance the malignant behaviour of tumour cells, including over-proliferation, metastasis and less apoptosis 35, 36. In human lung cancer, low expression of HSP27 is associated with better prognosis 37. However, the role of phosphorylated HSP27 in apoptosis remains ambiguous 24. In the present study, we observed that the expression of phosphorylated p38-MAPK in lung SCC was increased with upregulation of phosphorylated HSP27 after treatment with dioscin both *in vivo* and *in vitro*. The activation of p38-MAPK/HSP27 induced by the p38-MAPK activator Anisomycin caused the apoptosis of lung SCC cells. On the other hand, the p38-MAPK inhibitor SB203580 attenuated dioscin-induced apoptosis. Thus, we concluded that dioscin induces apoptosis through the p38-MAPK/HSP27 signalling pathway. Similar to our findings, activation of p38-MAPK/HSP27 induces brain endothelial cell apoptosis 24. A recent study found that activation of p38-MAPK/HSP27 suppresses stem cell-like properties in human non-small cell lung cancers, which indicates a new function of p38-MAPK in tumour suppression 38. To further investigate whether ROS regulates the p38-MAPK/HSP27 pathway in lung SCC cells, we treated the cells with NAC and found that NAC restrains dioscin-induced activation of p38-MAPK/HSP27. Above all, we illustrated that intracellular ROS regulated the activation of p38-MAPK/HSP27 in the dioscin-induced apoptosis of lung SCC cells *in vitro*.

## Conclusion

In summary, our findings demonstrate that dioscin facilitates ROS-induced apoptosis via the p38-MAPK/HSP27-mediated pathways in lung SCC cells and suggested that dioscin might be used as a potential anticancer agent in lung SCC treatment.

## Figures and Tables

**Figure 7:**
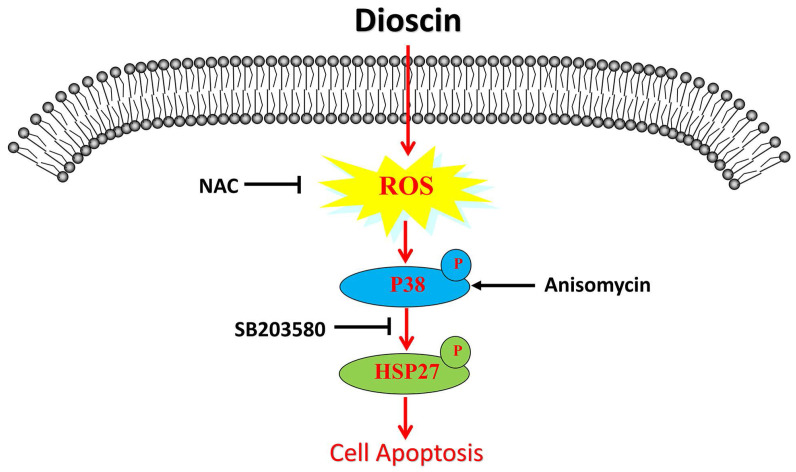
Schematic illustration of dioscin-induced apoptosis in lung squamous cell carcinoma.

## Abbreviations

SCC: squamous cell carcinoma
ROS: reactive oxygen species
NAC: N-acetyl-L-cysteine
HSP27: heat shock protein 27
FBS: fetal bovine serum
DMSO: dimethyl sulfoxide
DCFH‑DA: 2',7'‑dichlorodihydrofluorescein diacetate
DAPI: 4′,6-diamidino-2-phenylindole
EMT: epithelial-to-mesenchymal transition
Bcl2: B cell lymphoma 2
SHP2: SH2 domain-containing phosphatase-2
